# Evidence for Widespread Degradation of Gene Control Regions in Hominid Genomes

**DOI:** 10.1371/journal.pbio.0030042

**Published:** 2005-01-25

**Authors:** Peter D Keightley, Martin J Lercher, Adam Eyre-Walker

**Affiliations:** **1**School of Biological Sciences, University of EdinburghEdinburghUnited Kingdom; **2**Department of Biology and Biochemistry, University of BathBathUnited Kingdom; **3**Centre for the Study of Evolution and School of Life Sciences, University of SussexBrightonUnited Kingdom; University of California at BerkeleyUnited States of America

## Abstract

Although sequences containing regulatory elements located close to protein-coding genes are often only weakly conserved during evolution, comparisons of rodent genomes have implied that these sequences are subject to some selective constraints. Evolutionary conservation is particularly apparent upstream of coding sequences and in first introns, regions that are enriched for regulatory elements. By comparing the human and chimpanzee genomes, we show here that there is almost no evidence for conservation in these regions in hominids. Furthermore, we show that gene expression is diverging more rapidly in hominids than in murids per unit of neutral sequence divergence. By combining data on polymorphism levels in human noncoding DNA and the corresponding human–chimpanzee divergence, we show that the proportion of adaptive substitutions in these regions in hominids is very low. It therefore seems likely that the lack of conservation and increased rate of gene expression divergence are caused by a reduction in the effectiveness of natural selection against deleterious mutations because of the low effective population sizes of hominids. This has resulted in the accumulation of a large number of deleterious mutations in sequences containing gene control elements and hence a widespread degradation of the genome during the evolution of humans and chimpanzees.

## Abstract 2

A comparison of hominid and rodent lineages reveals that the gene control regions of hominids are not conserved and are accumulating mutations, suggesting widespread degradation of the hominid genome

## Introduction

Functionally important sequences are expected to evolve more slowly than neutrally evolving sequences. This is because long periods of selection for functional efficiency lead to sequences in which most advantageous mutations have already become fixed. The majority of new mutations in a sequence are then deleterious, because they perturb the highly adapted state. Studies of protein-coding DNA evolution have supported this expectation by showing that rates of amino acid substitution are substantially lower than rates of synonymous substitution in the majority of genes (e.g., [1]).

Recently there has been great interest in using sequence conservation to detect functionally important regions of the genome outside of protein-coding sequences. However, detecting conservation has proven difficult because noncoding DNA sequences often appear to be weakly conserved. In mammals, for example, more than 98.5% of the genome is believed to be intron or intergenic DNA [2], and at least 40% of this is composed of the remnants of transposable element insertions that are presumably decaying in a neutral fashion. Comparisons of the rates of evolutionary divergence between species have revealed evolutionary constraints in certain classes of intergenic DNA, particularly in DNA close to coding sequences [3], regions in which gene expression control motifs are believed to be prevalent. For example, on the basis of comparisons between rodent genomes, it has been suggested that there are at least as many selectively constrained sites outside, as within, protein-coding sequences [4,5]. Between mouse and rat, comparisons of rates of evolutionary divergence of non-protein-coding DNA imply that about 17% of sites in the 2 kb upstream and downstream from genes and in first introns are selectively constrained sites [6]. There is also evidence for highly conserved DNA sequences at locations distant from coding sequences [6,7,8,9,10,11].

However, the extent to which constraint in noncoding regions varies among species is unclear. In this paper, we investigate sequence conservation in introns and intergenic DNA in interspecific comparisons of mouse and rat (murids) and human and chimpanzee (hominids). To estimate the levels of constraint in segments of non-protein-coding DNA, we compare the observed numbers of substitutions to the number expected from substitution rates at linked sequences assumed to be neutrally evolving. Unexpectedly, we find that selective constraints are essentially absent in hominids in regions upstream of genes and in first introns, in contrast to murids, in which these regions are subject to moderate levels of constraint.

## Results/Discussion

### Selective Constraints in Hominids and Murids

We investigated the frequency of deleterious mutations and the pattern of sequence conservation in regions containing gene expression control sequences in hominids by compiling a dataset of 1,000 well-annotated, randomly chosen human genes. The genomic sequences upstream and downstream of each coding sequence and samples of their introns were aligned against the draft chimpanzee sequence. We compared the pattern of evolution in these hominid sequences against that in a previously compiled dataset of murid sequences [6]. Numbers of nucleotides sampled and other summary statistics for the sequences are shown in Table 1. As others have noted previously, intron sequences evolve slightly faster (10%–25%) than 4-fold degenerate synonymous sites, when hypermutable CpG dinucleotides are excluded. (CpG dinucleotides are more frequent in coding sequences than intergenic DNA.) This could imply the presence of selection on synonymous sites [12,13]. Of the genomic sequences surveyed, intron sequences, other than intron 1, appear to be the fastest-evolving sequences in mammalian genomes and are therefore used as our neutral standard.

**Table 1:**
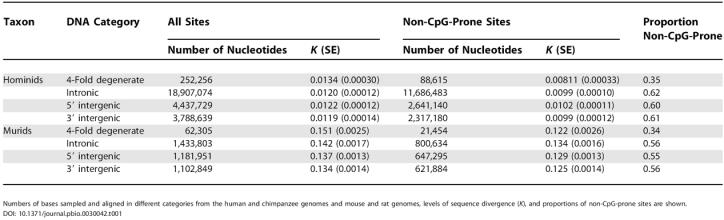
Summary Statistics for Sequences Sampled from Hominid and Murid Genomes

Numbers of bases sampled and aligned in different categories from the human and chimpanzee genomes and mouse and rat genomes, levels of sequence divergence (*K*), and proportions of non-CpG-prone sites are shown

We calculated levels of selective constraint in blocks of 500 bp, averaged over loci, using the aligned human–chimpanzee and mouse–rat genome sequence datasets. We corrected our constraint estimates for the decline in GC content that appears to be occurring in most mammalian genomes, including hominids and murids [14], although this correction made little difference, since the GC content of the flanking regions is similar to that of introns. Because different parts of a sequence can differ markedly in their frequency of CpG dinucleotides, and most CpG dinucleotides are hypermutable and are saturated by nucleotide substitutions between mice and rats, we excluded potentially CpG-prone sites by excluding sites preceded by C or followed by G. Simulation results ([6]; D. J. Gaffney and P. D. Keightley, unpublished data) indicated that failure to exclude CpG-prone sites leads to biased estimates of constraint.

**Figure 1:**
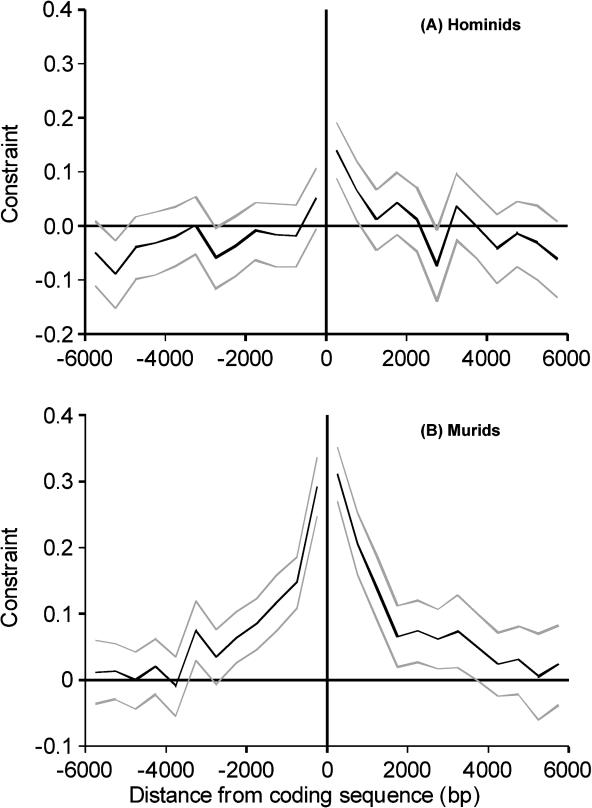
Selective Constraint Plotted against Distance from the Coding Sequence in 5′ Flanking Regions and 3′ Flanking Regions Results for (A) hominids and (B) murids. The 5′ flanking regions are shown left of the origin, and 3′ flanking regions, right. Segments of 500 bases starting from the start or stop codon were analyzed. Bootstrap 95% confidence limits are shown in light grey.

**Figure 2:**
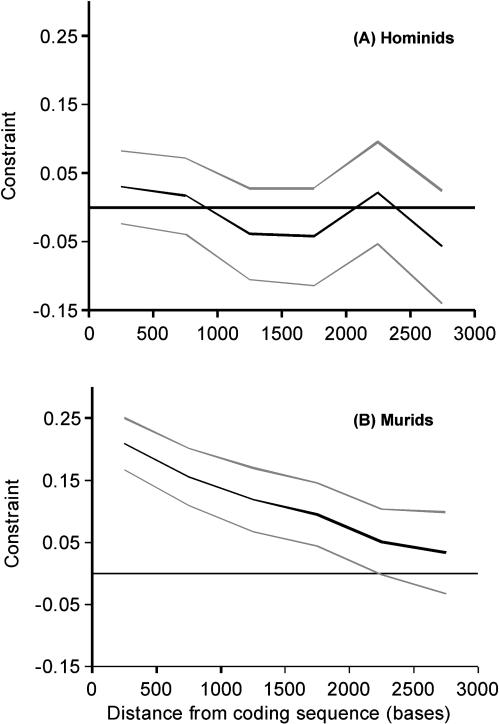
Selective Constraint at the 5′ End of First Introns Plotted against Distance from the Intron Start Results for (A) hominids and (B) murids. Segments of 500 bases starting from the 5′ end of intron 1 were analyzed. Bootstrap 95% confidence limits are shown in light grey. Constraint at the 3′ end of first introns is nonsignificant and close to zero in both hominids and murids.

Selective constraints in hominid sequences in the regions 6,000 bp upstream and downstream of coding sequences and in the 5′ regions of first introns are clearly far lower than in murid sequences (Figures 1 and 2). In particular, for the first 2,000 bp of the 5′ flanking region and the first 2,000 bp at the 5′ end of intron 1, constraint estimates (± standard error) are 0.0016 (± 0.019) and −0.0029 (± 0.019), respectively, for hominids, and are 0.17 (± 0.016) and 0.16 (± 0.018) for murids (Table 2). The differences in constraint between hominids and murids in these regions are therefore about six times larger than the standard errors of differences between constraint estimates. Constraint is significantly different from zero near the 3′ end of hominid coding sequences, but still more than 50% lower than in murids (Table 2). The standard errors for these estimates are very similar in murids and hominids, indicating that we have similar power to detect constraint in the two datasets. Differences in mean levels of constraint between hominids and murids are somewhat smaller if all nucleotide sites (including CpG-prone sites) are analyzed (Table 2), but constraint in 5′ flanking regions and first introns is still very low in hominids (approximately 0.03) and significantly higher in murids. Such estimates of constraint are likely to be downwardly biased in murids, owing to saturation at CpG dinucleotides, giving an underestimate of the substitution rate in introns. This may be partly offset by the fact that some gene control regions are in unmethylated CpG islands, which would tend to increase estimates of constraint in both hominids and murids.

**Table 2:**
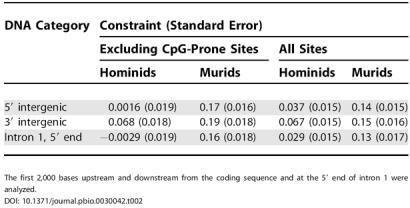
Constraint in Hominids and Murids Calculated for Datasets Excluding CpG-Prone Sites and for Datasets Including All Sites

The first 2,000 bases upstream and downstream from the coding sequence and at the 5′ end of intron 1 were analyzed

There are a number of possible explanations for the apparent absence of constraint in hominid 5′ flanking and first intron sequences (and for the lower constraint in the 3′ flanking region), which we examine below.

### Sequencing Errors, Pseudogenes, and Genome Reorganization

Because the mean sequence divergence of hominids is an order of magnitude lower than that of murids, sequencing errors are expected to disproportionally downwardly bias estimates of constraint in hominids. To estimate the extent of sequencing error in our data, we investigated how many conserved intron splice junctions had been mis-sequenced in the chimpanzee—the chimpanzee sequence has been produced and assembled without reference to the human sequence, so this should provide an unbiased estimate of the error rate. We observed 25 differences in 21,048 intronic splice donor/acceptor nucleotides, giving a maximum error rate (ɛ) of 1.19 × 10^−3^. This is expected to reduce constraint by approximately one-tenth since the relationship between the true level of constraint (*C_true_*) and observed level (*C_obs_*) is *C_obs_ = C_true_*/(1 + ɛ/*k*), and the human–chimp divergence (*k*) is approximately 1%. If the true levels of constraint in the 5′, 3′, and intron 1 sequences closest to hominid genes were 30%, as we find in murids, the observed constraint in hominids would be 27%. Sequencing errors can therefore explain only a small proportion of the difference in constraint between hominids and murids. Further evidence that our data do not have unusually high rates of sequencing errors is that the divergences (see Table 1) are very similar to values that have been reported previously [12,15,16,17]. The results are also unlikely to be explained by polymorphism since levels of diversity in hominids are about 0.1% [18].

A second possibility that could explain the differences in constraint between hominids and murids is that our dataset of hominid genes contains large numbers of pseudogenes. However, several lines of evidence argue against this. First, the genes are well annotated and contain no stop codons. Second, the intronic splice donor/acceptor nucleotides are highly conserved. Third, the exons of our gene sample show strong conservation: constraint at second positions of codons estimated in the same manner as for noncoding sequences is 0.750 ± 0.013. And finally, a very substantial proportion of genes would have to be pseudogenes to explain our data. For example, to reduce the constraint by one-half would require 50% of the hominid genes to be pseudogenes.

A third possibility is that the lower constraints in hominid 5′ flanking and first intron regions could be a consequence of a reorganization of gene regulation such that murids have a concentration of regulatory sequences in 5′ regions and in first introns and hominids have regulatory sequences concentrated in introns outside intron 1. However, two lines of evidence suggest that this is unlikely. First, there is remarkable conservation of syntenic blocks [19] and per locus intron/exon number [4,5] between murids and human. Second, the vast majority of known mammalian gene expression regulatory regions are situated within 2 kb of promoters [4].

### Adaptive Evolution

**Figure 3:**
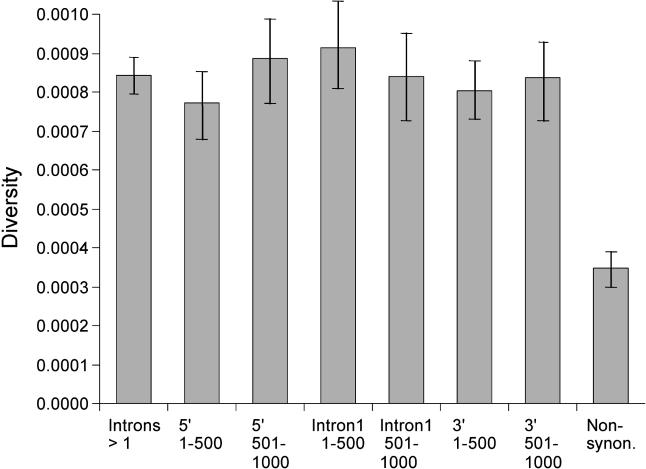
Mean Nucleotide Diversity in Human Intergenic DNA in Blocks of 500 Bases Upstream and Downstream of Genes Data shown separately for first introns, introns excluding first introns (“Introns > 1”), and nonsynonymous sites. 95% confidence limits are indicated.

The lower level of constraint in hominids could be due to higher rates of adaptive substitution in the 5′, 3′, and first intron regions in hominids, masking constraint on other sites. If this is the case then we expect reduced nucleotide diversity in the 5′, 3′, and first intron regions for two reasons. First, the level of diversity in a region is largely determined by the number of effectively neutral mutations, because adaptive substitutions contribute little to polymorphism. This implies that levels of polymorphism will be lower if some sites are subject to constraint. Second, levels of diversity are expected to be lower in regions undergoing adaptive substitution because adaptive substitutions can remove variation by genetic hitchhiking [20]. To test for reduced levels of variation, we analyzed single nucleotide polymorphism data from the 5′ flanking, 3′ flanking, and intron sequences of 305 human genes compiled from the Environmental Genome Project (http://www.niehs.nih.gov/envgenom/home.htm). This dataset was chosen because it represents the most extensive and consistently sampled database of human single nucleotide polymorphisms. The genes were sampled as being “environmentally responsive,” and are therefore not a random sample, but they show average levels of constraint in all regions similar to those of our sample of 1,000 hominid genes. The analysis revealed no reduction in diversity in the 5′, 3′, and intron 1 regions when compared to the levels of diversity in intron sequences outside first introns (Figure 3). In contrast, the level of diversity is significantly lower at nonsynonymous sites. To test the adaptive hypothesis further, we aligned the polymorphism dataset of 305 human genes against the chimpanzee genome sequence, measured divergence, and tested for adaptive evolution by an extension of the McDonald–Kreitman test [21] under the assumption that introns other than intron 1 are evolving neutrally (Table 3). There was no evidence of adaptive evolution in these tests or in tests in which polymorphisms segregating at less than 10% were excluded—excluding rare polymorphisms has the effect of removing any slightly deleterious mutations that might be segregating in the 5′, 3′, and intron 1 sequences [22]. Interestingly the estimate of proportion of substitutions driven by adaptive evolution (α) was significantly negative for the 3′ flanking region. This is likely due to the segregation of slightly deleterious mutations, which is consistent with the low but significant level of constraint we observed in this region.

**Table 3:**
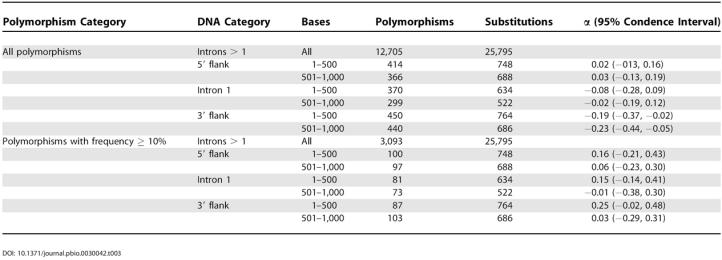
Tests of Adaptive Evolution for 5′, 3′, and Intron 1 Sequences in Hominids

### Fixation of Mildly Deleterious Mutations

Two lines of evidence suggest that many mutations that affect gene expression may be under only weak purifying selection. First, sequences involved in gene regulation often appear to evolve rapidly [23]. Second, the rate of divergence in gene expression of primate genes is as fast as that of expressed pseudogenes [24]. These observations suggest another explanation for the low constraint in hominids: selection on mutations in the 5′ and 3′ flanking and intron 1 sequences that affect gene expression may be ineffective in hominids, since hominids have low effective population sizes (*N_e_*)*.* Based on polymorphism data and estimates of nucleotide mutation rates (*u*), estimates of human and chimpanzee *N_e_* are typically in the range 10,000–30,000 for nuclear sequences [25,26,27], and this is likely to have been the case for much of their evolution, since the ancestral *N_e_* for both species is estimated to be approximately 20,000 [28]. Unfortunately, we have little data from murids with which to estimate effective population sizes. A recent survey of nucleotide diversity in Mus musculus domesticus yielded an estimate of 4*N_e_u* of 0.0054 [29]. Combining this with as estimate of the nucleotide mutation rate of between 1.67 × 10^−9^ and 2.98 × 10^−9^ [25], we estimate *N_e_* for the house mouse to be between 450,000 and 810,000. The fate of a deleterious mutation depends on the product *N_e_s*; if *N_e_|s|* > 1, then the fixation probability for a deleterious mutation starts to become appreciable. Therefore, deleterious mutations, whose strength of selection falls within the range 1/*N_e_*(murids) < |*s|* < 1/*N_e_*(hominids), will tend to be removed by natural selection in murids, but can drift to fixation in hominids. Under the assumption that selection coefficients against deleterious mutations are equivalent in all taxa, the levels of selective constraint in noncoding DNA of murids and hominids imply that approximately 83%, 17%, and 0% of mutations in the first 2,000 bp of 5′ flanking DNA and intron 1 have a strength of selection such that |*s*| < 1/*N**_e_***(rodents), 1/*N_e_*(rodents) < |*s*| < 1/*N_e_*(hominids) and |*s*| > 1/*N_e_*(hominids), respectively. For the the first 2,000 bp of 3′ flanking DNA we estimate that about 81% of mutations have |*s*| < 1/*N_e_*(rodents), 12% are in the range 1/*N_e_*(rodents) < |*s*| < 1/*N_e_*(hominids) and 7% have |*s*| >1/*N_e_*(hominids). In contrast to noncoding DNA, the fraction of slightly deleterious mutations fixed in hominid coding sequences is quite low. Constraint estimates at second codon positions outside CpG-prone sites in hominids and murids are 0.750 ± 0.016 and 0.900 ± 0.0085, respectively. Taking into account sequencing errors in hominids, the predicted “true” constraint value in hominids is 0.84, and this implies that only about 6% of mutations are in the slightly deleterious class.

### Gene Expression Divergence

The lower level of constraint in hominid 5′, 3′, and intron 1 sequences leads to a testable prediction about the evolution of gene expression. Since the flanking regions of genes contain a high concentration of *cis-*acting gene control sequences [4], we expect gene expression to be evolving more rapidly in hominids than in murids, relative to the rate of neutral sequence evolution (i.e., the rate of mutation). To test this prediction, we used the microarray data of Enard et al. [30], who examined gene expression profiles in brain and liver tissue for humans, chimpanzees, M. domesticus, and M. spretus. From an analysis of 3,801 orthologous genes across all four species, we found that levels of divergence in expression between human and chimp are very similar to levels of divergence in expression between the two mouse species (Table 4). At the same time, the level of nucleotide divergence in introns, *K_i_*, between the two hominid species is about 55% that of the mouse species (Table 4). Thus, when measured relative to the level of intron nucleotide divergence, the divergence in gene expression *d* is almost 1.8-fold higher in hominids than it is in murids. This acceleration is significant both for liver (hominid/murid ratio of *d*/*K_i_*, 1.71; 95% confidence interval, 1.46–2.12) and for brain (hominid/murid ratio of *d*/*K_i_*, 1.79; 95% confidence interval, 1.53–2.21).

**Table 4:**
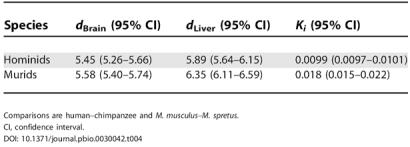
Expression Divergence (*d*) and Intron Nucleotide Divergence outside CpG-Prone Sites (*K_i_*) for Hominids and Murids

Comparisons are human–chimpanzee and M. musculus–M. spretus

CI, confidence interval

As demonstrated in an analysis across different primate species, substantial increases in expression distances are still observed when going beyond the evolutionary distances examined here [30]. Thus, our finding of similar expression distances between the two species pairs cannot be due to expression distances reaching saturation. Because the Euclidean expression distances in Table 4 are of comparable magnitude, our conclusions are independent of the exact relationship between expression divergence and sequence divergence; similar results are obtained when using the mean squared gene-wise difference in log-expression (data not shown), as used, for example, by Khaitovich et al. [24].

A potential source of error in this analysis is the use of microarrays designed for humans on chimpanzee samples, and of microarrays designed for M. musculus on M. spretus samples. Sequence differences between the species will lead to lower hybridization efficiencies in chimpanzee and M. spretus, and will consequently exaggerate expression distances. However, this problem will be more pronounced in the mouse comparisons, since sequence divergence between M. musculus and M. spretus is higher than between human and chimpanzee. Thus, this would bias our results towards higher rates of gene expression evolution in mice, making our test conservative.

### Conclusion

The lack of conservation of regions containing expression control sequences demonstrated above is consistent with the observation that the vast majority of Mendelian genetic disease mutations are located in coding sequences [31]. Our results have a number of interesting repercussions. The virtual absence of constraint in 5′ flanking regions and in 5′ regions of first introns, in which the majority of mammalian gene expression control sequences are believed to reside [4], implies that there has been widespread degradation of regions containing gene control sequences in hominids. We estimate that humans and chimpanzees have accumulated approximately 140,000 slightly deleterious mutations each, mutations that would have been eliminated by selection in murids. These mutations have small effects, since it can be inferred that they have selection coefficients less than 1/*N_e_* for hominids, i.e., less than 10^−4^. It should be noted that it is unlikely that the mutation accumulation is due to a recent relaxation of natural selection in humans due to an improvement in our living conditions [32], since the time of this improvement is short relative the overall length of human evolution. We would not expect the decline in fitness to continue indefinitely, since the absolute strength of selection on new mutations, both advantageous and deleterious, may increase as fitness declines [33]. Furthermore, this accumulation of deleterious mutations may have been compensated in part by adaptive substitutions in gene expression control regions and elsewhere in the genome.

We have also demonstrated that gene expression evolution is significantly accelerated in hominid brain and liver compared to the respective murid tissues. This result has important implications for theories of neutral gene expression evolution [24,34]. First, our results are consistent with the view that most variation in gene expression level found between human alleles is neutral [30,35,36]. However, the difference in expression divergence, relative to nucleotide divergence, between hominid and murid genomes implies that the proportion of gene expression changes that are under natural selection varies between different lineages, and that many of the mutations that affect gene expression in murids may in fact be under selection. Consequently, the strict notion of a gene expression clock [24], like the molecular clock, may only apply within closely related species. This is true irrespective of whether time is measured in units of sequence divergence (See Table 1) or in years: the divergence time between human and chimp is approximately 6 million years, while between M. musculus and M. spretus it is approximately 1.8 million years [24], so the absolute rate of gene expression divergence is much slower in hominids than murids, whilst it is substantially faster compared to the rate of neutral sequence evolution.

The importance of effective population size in influencing the organization and complexity of genomes has recently been highlighted [37]. Our findings support the idea that microevolutionary processes are also strongly influenced by population size, and are evidence for the nearly neutral model of molecular evolution [38] in mammalian genomes.

## Materials and Methods

### 

#### Sampling of hominid genomic sequences

DNA sequences of 1,000 annotated loci were compiled from the reference sequence (build 33) of the human genome. In a preliminary analysis of a smaller dataset, we determined that such a dataset of 1,000 loci would provide standard errors on constraint estimates of less than 2%. We randomly sampled loci by the criterion that each record contained the description of at least one mRNA. We extracted all exons, up to eight introns, including first, second, last, and second last introns, and up to 6 kb of intergenic DNA 5′ and 3′ from the start and stop codon, respectively. Intergenic DNA was extracted up to the midpoint between the sampled coding sequence and the start or stop codon of the following or preceding locus in the genomic contig. We extracted complete introns if they were less than 30 kb in length, otherwise the first and last 10 kb. For more than 80% of the hominid loci sampled, the 6-kb 5′ region includes all annotated untranslated exons and introns.

We used reciprocal best-hits BLAST [39] to identify sequences orthologous to the human sequences in the reference assembly of the whole genome shotgun assembly of the chimpanzee genome. If a DNA segment exceeded 2 kb in length, this was subdivided into approximately 1-kb segments for analysis via BLAST. Sequences were aligned using MCALIGN [40] under a model of indel evolution appropriate to hominid intronic DNA. Parts of the chimp genome are of relatively low sequencing coverage, and errors in the assembly are expected. We therefore masked off sequence segments containing more than ten mismatches in a stretch of 100 bp and more than five mismatches in a stretch of 25 bp. Assuming independently distributed substitutions, the first level of nonhomology is expected almost never to appear by chance in our dataset, and the second level is expected to occur approximately four times in the approximately 27 Mb surveyed (see Table 1).

#### Sampling of murid sequences

Orthologous genes from 300 well-annotated loci were randomly sampled from the whole-genome assemblies of mouse and rat from GenBank. Loci were chosen for which annotation evidence included at least one complete mRNA sequence in both species. Further details of the sampling are given in [6]. Coding sequences, a sample of up to three introns (including first and last introns), and up to 6 kb of intergenic DNA 5′ and 3′ from the start and stop codon, respectively, were extracted from both species. Sequences were aligned by MCALIGN [40] using a model of indel evolution appropriate to rodent intronic DNA as described previously [6].

#### Calculation of evolutionary constraint

We estimated selective constraints (*C*) for each of the above categories of sites by comparing the observed numbers of substitutions (*O*) with numbers expected (*E*) if substitution rates were equal to that of a class of putatively neutral sites. In this analysis, these putatively neutral sites were intronic sites excluding intronic splice control regions (bases 1–6 and 1–16 at the 5′ and 3′ ends, respectively) and first introns, since these show evidence of moderate selective constraint [6]. If the effect of higher substitution rates within the CpG dinucleotide context is removed, mean substitution rates in both murid and hominid lineages are somewhat higher at introns than at 4-fold degenerate sites ([5]; see Table 1); synonymous sites are believed to be under weak selection in mammals [1]. The level of evolutionary constraint for a specific category of sites in *n* loci is



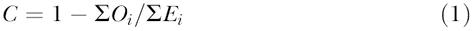



[41]. Constraint was calculated excluding nucleotides preceded by C or followed by G. Such CpG-susceptible sites have a high probability of being part of a hypermutable CpG dinucleotide, which approach saturation between mouse and rat, and have multiple substitutions sufficiently frequently between human and chimp as to induce bias in estimating substitution rates. The mutation process at microsatellite loci differs radically from that for nucleotide substitutions, so these were excluded from the analysis [6].

A nonequilibrium model of base composition evolution was used to calculate *E,* as described previously [42], assuming an equilibrium GC content of 0.4. For coding sequences, constraint was calculated for second positions of codons, where substitutions always lead to an amino acid change, using intronic sequences as the neutral reference. In noncoding DNA, constraint was calculated for blocks, typically of 500 bp, and averaged over loci using equation 1. Standard errors and confidence intervals were computed by bootstrapping over loci.

The number of slightly deleterious mutations fixed in hominids was calculated from the product of 25,000 (genes) × 4,000 bp (of 5′ and intron 1 sequence) × 0.17 (difference in constraint between murids and hominids) × 0.012/2 (human–chimp divergence/2) + 25,000 (genes) × 2,000 bp (of 3′ sequence) × 0.12 (difference in constraint between murids and hominids) × 0.012/2 = 138,000.

#### Test of adaptive evolution

Single nucleotide polymorphism data for 335 genes were compiled from the Environmental Genome Project (http://www.niehs.nih.gov/envgenom/home.htm). Of these, 305 had more than two intron sequences, and were suitable for further analysis. The sequences were aligned against the chimpanzee genome sequence, as described above, and the number of substitutions estimated by counting the number of differences, with no correction made for multiple substitutions (humans and chimps are sufficiently close as to make corrections for multiple substitutions unnecessary, particularly when CpG dinucleotides are excluded). We only considered sites that were not preceded by C or followed by G to maintain consistency with the other analyses reported here. For each gene we calculated the numbers of 5′ (or 3′ or intron 1) substitutions (*D_n_*) and polymorphisms (*P_n_*), along with the equivalent figures for the other introns, which acted as our neutral standard (respectively *D_i_* and *P_i_*). To test for adaptive evolution we estimated the proportion of substitutions that were driven by adaptive evolution using the method of Smith and Eyre-Walker [21]:



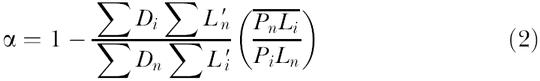



where *L_n_* and *L_i_* are the numbers of putatively selected and intron sites, respectively. Note that the number of sites appears in this formula because the number of sites for the polymorphism and divergence data are slightly different, since not all the sequence could be aligned against the chimpanzee genome. Figures for the divergence data are indicated by a prime. To obtain confidence limits for α, we bootstrapped the data by gene.

#### Gene expression data

Affymetrix oligonucleotide microarray data for brain cortex and liver tissue samples from three individuals each of human, chimpanzee, M. musculus, and M. spretus were obtained from Enard et al. [30]. To have comparable data from all species, only the first replicate of each human and chimp was used. Raw hybridization intensities were converted to expression levels using the Affymetrix MAS5 function as implemented in the BioConductor package [43], and then log_2_-transformed. The primate expression data were restricted to probe sets contained in both the HG-U95Av2 microarray (used for liver) and the HG-U95A microarray (used for cortex).

We restricted the expression analysis to orthologous genes as follows. From Ensembl (www.ensembl.org), we obtained a mapping of probe sets to Ensembl gene IDs, and a list of human–mouse orthologs. If more than one probe set matched the same Ensembl gene, we averaged expression levels over all probe sets for this gene. We retained only one-to-one orthologs (i.e., genes where human and mouse IDs uniquely match each other), further requiring that each sequence cover at least 70% of the other. Expression matrices for the individual experiments were scaled to the same mean. All mouse experiments (and, separately, all primate experiments) were then normalized relative to each other by means of a quantile normalization [44]. For each species/tissue combination, these normalized expression values were averaged over the three individuals, resulting in expression vectors for 3,801 genes orthologous between human and mouse.

Expression distances between species were calculated as Euclidean distances between expression vectors. Bootstrap analysis (resampling of genes; 1,000 datasets) was used to estimate standard errors (standard deviation of bootstrap distances) and confidence intervals (2.5% and 97.5% quantiles of bootstrap distances).

We compared expression differences to intronic nucleotide divergence levels calculated without correction for multiple substitutions, excluding CpG-prone sites as described above. We analyzed the complete 1,000 gene chimp–human intronic dataset described above and a dataset of 39 introns from 24 orthologous loci of M. spretus and M. domesticus, compiled from GenBank. In order to obtain confidence intervals for the ratio of expression divergence *d* over sequence divergence *K_i_*, we used a bootstrap analysis of 1,000 datasets, each combining *d* values obtained from resampling genes from the expression analyses, and *K_i_* values obtained from resampling genes from the divergence analyses.

## References

[pbio-0030042-b01] Graur D, Li WH (1999). Fundamentals of molecular evolution, 2nd ed.

[pbio-0030042-b02] International Human Genome Sequencing Consortium (2001). Initial sequencing and analysis of the human genome. Nature.

[pbio-0030042-b03] Duret L, Dorkeld F, Gautier C (1993). Strong conservation of non-coding sequences during vertebrates evolution: Potential involvement in post-transcriptional regulation of gene expression. Nucleic Acids Res.

[pbio-0030042-b04] Mouse Genome Sequencing Consortium (2002). Initial sequencing and comparative analysis of the mouse genome. Nature.

[pbio-0030042-b05] Rat Genome Sequencing Project Consortium (2004). Genome sequence of the Brown Norway rat yields insights into mammalian evolution. Nature.

[pbio-0030042-b06] Keightley PD, Gaffney DJ (2003). Functional constraints and frequency of deleterious mutations in non-coding DNA of rodents. Proc Natl Acad Sci U S A.

[pbio-0030042-b07] Jareborg N, Birney E, Durbin R (1999). Comparative analysis of noncoding regions of 77 orthologous mouse and human gene pairs. Genome Res.

[pbio-0030042-b08] Frazer KA, Sheehan JB, Stokowski RP, Chen XY, Hosseini R (2001). Evolutionarily conserved sequences on human chromosome 21. Genome Res.

[pbio-0030042-b09] Dermitzakis ET, Reymond A, Lyle R, Scamuffa N, Ucla C (2002). Numerous potentially functional but non-genic conserved sequences on human chromosome 21. Nature.

[pbio-0030042-b10] Dermitzakis ET, Reymond A, Scamuffa N, Ucla C, Kirkness E (2003). Evolutionary discrimination of mammalian conserved non-genic sequences (CNGs). Science.

[pbio-0030042-b11] Bejerano G, Pheasant M, Makunin I, Stephen S, Kent WJ (2004). Ultraconserved elements in the human genome. Science.

[pbio-0030042-b12] Hellmann I, Zollner S, Enard W, Ebersberger I, Nickel B (2003). Selection on human genes as revealed by comparisons to chimpanzee cDNA. Genome Res.

[pbio-0030042-b13] Urrutia AO, Hurst LD (2003). The signature of selection mediated by expression on human genes. Genome Res.

[pbio-0030042-b14] Duret L, Semon M, Piganeau G, Mouchiroud D, Galtier N (2002). Vanishing GC-rich isochores in mammalian genomes. Genetics.

[pbio-0030042-b15] Chen FC, Li WH (2001). Genomic divergences between humans and other hominoids and the effective population size of the common ancestor of humans and chimpanzees. Am J Hum Genet.

[pbio-0030042-b16] Ebersberger I, Metzler D, Schwarz C, Pääbo S (2002). Genome-wide comparison of DNA sequences between humans and chimpanzees. Am J Hum Genet.

[pbio-0030042-b17] Watanabe H, Fujiyama A, Hattori M, Taylor TD, Toyoda A (2004). DNA sequence and comparative analysis of chimpanzee chromosome 22. Nature.

[pbio-0030042-b18] Li WH, Sadler LA (1991). Low nucleotide diversity in man. Genetics.

[pbio-0030042-b19] Gregory SG, Sekhon M, Schein J, Zhao SY, Osoegawa K (2002). A physical map of the mouse genome. Nature.

[pbio-0030042-b20] Maynard Smith J, Haigh J (1974). The hitch-hiking effect of favorable genes. Genet Res.

[pbio-0030042-b21] Smith NGC, Eyre-Walker A (2002). Adaptive protein evolution in Drosophila. Nature.

[pbio-0030042-b22] Fay J, Wycoff GJ, Wu CI (2001). Positive and negative selection on the human genome. Genetics.

[pbio-0030042-b23] Ludwig MZ, Kreitman M (1995). Evolutionary dynamics of the enhancer region of even-skipped in Drosophila. Mol Biol Evol.

[pbio-0030042-b24] Khaitovich P, Weiss G, Lachmann M, Hellmann I, Enard W (2004). A neutral model of transcriptome evolution. PLoS Biol.

[pbio-0030042-b25] Eyre-Walker A, Keightley PD, Smith NGC, Gaffney D (2002). Quantifying the slightly deleterious model of molecular evolution. Mol Biol Evol.

[pbio-0030042-b26] Yu N, Fu YX, Li WH (2002). DNA polymorphism in a worldwide sample of human X chromosomes. Mol Biol Evol.

[pbio-0030042-b27] Yu N, Jensen-Seaman MI, Chemnick L, Kidd JR, Deinard AS (2003). Low nucleotide diversity in chimpanzees and bonobos. Genetics.

[pbio-0030042-b28] Rannala B, Yang Z (2003). Bayes estimation of species divergence times and ancestral population sizes using DNA sequences from multiple loci. Genetics.

[pbio-0030042-b29] Ideraabdullah FY, de la Casa-Esperon E, Bell TA, Detwiler DA, Magnuson T (2004). Genetic and haplotype diversity among wild-derived mouse inbred strains. Genome Res.

[pbio-0030042-b30] Enard W, Khaitovich P, Klose J, Zollner S, Heissig F (2002). Intra- and interspecific variation in primate gene expression patterns. Science.

[pbio-0030042-b31] McKusick VA (1998). Mendelian inheritance in man: A catalog of human genes and genetic disorders, 12th ed.

[pbio-0030042-b32] Crow JF (1997). The high spontaneous mutation rate: Is it a health risk?. Proc Natl Acad Sci U S A.

[pbio-0030042-b33] Akashi H (1995). Inferring weak selection from patterns of polymorphism and divergence at “silent” sites in Drosophila DNA. Genetics.

[pbio-0030042-b34] Yanai I, Graur D, Ophir R (2004). Incongruent expression profiles between human and mouse orthologous genes suggest widespread neutral evolution of transcription control. OMICS.

[pbio-0030042-b35] Yan H, Yuan W, Velculescu VE, Vogelstein B, Kinzler KW (2002). Allelic variation in human gene expression. Science.

[pbio-0030042-b36] Whitney AR, Diehn M, Popper SJ, Alizadeh AA, Boldrick JC (2003). Individuality and variation in gene expression patterns in human blood. Proc Natl Acad Sci U S A.

[pbio-0030042-b37] Lynch M, Conery JS (2003). The origins of genome complexity. Science.

[pbio-0030042-b38] Ohta T (1973). Slightly deleterious mutant substitutions in evolution. Nature.

[pbio-0030042-b39] Altschul SF, Madden TL, Schaffer AA, Zhang J, Zhang Z (1997). Gapped BLAST and PSI-BLAST: A new generation of protein database search programs. Nucleic Acids Res.

[pbio-0030042-b40] Keightley PD, Johnson T (2004). MCALIGN: Stochastic alignment of noncoding DNA sequences based on an evolutionary model of sequence evolution. Genome Res.

[pbio-0030042-b41] Eyre-Walker A, Keightley PD (1999). High genomic deleterious mutation rates in hominids. Nature.

[pbio-0030042-b42] Halligan DL, Eyre-Walker A, Andolfatto P, Keightley PD (2004). Patterns of evolutionary constraints in intronic and intergenic DNA of *Drosophila*. Genome Res.

[pbio-0030042-b43] Gentleman RC, Carey VJ, Bates DM, Bolstad B, Dettling M (2004). Bioconductor: Open software development for computational biology and bioinformatics. Genome Biol.

[pbio-0030042-b44] Bolstad BM, Irizarry RA, Astrand M, Speed TP (2003). A comparison of normalization methods for high density oligonucleotide array data based on variance and bias. Bioinformatics.

